# SP1/AKT/FOXO3 Signaling Is Involved in miR-362-3p-Mediated Inhibition of Cell-Cycle Pathway and EMT Progression in Renal Cell Carcinoma

**DOI:** 10.3389/fcell.2020.00297

**Published:** 2020-05-05

**Authors:** Hejia Zhu, Song Wang, Haixiang Shen, Xiangyi Zheng, Xin Xu

**Affiliations:** Department of Urology, First Affiliated Hospital, School of Medicine, Zhejiang University, Hangzhou, China

**Keywords:** miR-362-3p, SP1, FOXO3, renal cell carcinoma, microRNA

## Abstract

Emerging evidence has indicated that dysregulation of miR-362-3p is involved in the initiation and progression of several types of human cancers. However, the molecular mechanism of miR-362-3p in renal cell carcinoma (RCC) is still not completely clear. In this study, we found that miR-362-3p was frequently down-regulated in human RCC tissues. Overexpression of miR-362-3p in RCC cells significantly suppressed the proliferation, cell cycle and motility *in vitro* and *in vivo* via regulating AKT/FOXO3 signaling. We further confirmed that SP1 was a direct target of miR-362-3p. Knockdown of SP1 expression by a small interfering RNA (siRNA) phenocopied the effect of miR-362-3p overexpression in RCC cells. In conclusion, the current results provide evidence for the role of miR-362-3p in the pathogenesis of RCC and thus miR-362-3p may serve as an attractive candidate for RCC therapy.

## Introduction

Renal cell carcinoma (RCC) is the sixth most common cancer in males and tenth in females worldwide (Siegel et al., 2018), with an estimated 338 thousand new cases and 144 thousand deaths annually (Ferlay et al., 2015). Although substantial improvements have been achieved for RCC therapeutic strategies in recent years, RCC remains one of the most lethal urological malignancies (Capitanio et al., 2019). Therefore, it is urgently warranted to identify novel molecular biomarkers to improve the clinical outcomes of RCC patients.

MicroRNAs (miRNAs) are a conserved class of small non-coding RNAs (19–24 nucleotides) that regulate target gene expression by inducing mRNA degradation and/or suppressing mRNA translation (Bartel, 2004). Emerging evidence has demonstrated that miRNAs play a crucial role in tumorigenicity and progression in human cancers (Croce, 2009; Wang et al., 2018). For example, miR-362-3p has been reported to inhibit proliferation, migration and invasion in breast cancer and colorectal cancer, and downregulation of miR-362-3p is associated with a poor prognosis (Christensen et al., 2013; Kang et al., 2016). AKT/FOXO3 signaling regulates a variety of cellular processes including proliferation, cell cycle, apoptosis, epithelial-mesenchymal transition (EMT) and DNA damage (Liu et al., 2018). In this study, we found that miR-362-3p was down-regulated in RCC tissues and functioned as a tumor suppressor via regulating AKT/FOXO3 signaling pathway. In addition, SP1 was identified as a direct target of miR-362-3p. SP1 was considered a general transcription factor that was required for transcription of a large number of genes related with in metabolism, cell proliferation/growth, and cell death (Beishline and Azizkhan-Clifford, 2015). For example, acetylated SP1 was reported to be able to suppress PTEN expression via binding to PTEN promoter and recruitment of HDAC1 and promote cancer cell migration and invasion (Kou et al., 2013). Down-regulation of SP1 could inhibit cell proliferation, clonogenicity, and the expressions of stem cell markers in nasopharyngeal carcinoma (Zhang et al., 2014).

## Materials and Methods

### Reagents and Transfection

The miR-362-3p mimic (named miR-362-3p), the small interfering RNA targeting human SP1 mRNA (named siSP1), the negative control duplex (named NC) with no significant homology to any known human sequences were used for functional studies. The oligonucleotides are shown in Table 1. RNA transfection was performed using Lipofectamine 2000 reagents (Invitrogen, United States) according to the manufacturer’s protocol.

**TABLE 1:** The oligonucleotides used in this study.

Name*	Sequence (5′- > 3′)
miR-362-3p mimic (sense)	AACACACCUAUUCAAGGAUUCA
NC (sense)	ACUACUGAGUGACAGUAGA
siSP1 (sense)	GGAUGGUUCUGGUCAAAUATT
miR-362-3p F	AACACACCTATTCAAGGATTCA
U6 F	TGCGGGTGCTCGCTTCGGCAGC
SP1 F	GTGGAGGCAACATCATTGCTG
SP1 R	GCCACTGGTACATTGGTCACAT
GAPDH F	AAGGTGAAGGTCGGAGTCA
GAPDH R	GGAAGATGGTGATGGGATTT
SP1-utr-wt F	CGTGTGTGTGTGTGTGTGTGTGTGTGTGTGTGTGTGTAA TCTGTTAGGTTGG
SP1-utr-wt R	TCGACCAACCTAACAGATTACACACACACACACACACAC ACACACACACACACACGAGCT
SP1-utr -mut F	CGTGTGTGTGTGTGTGTGTGTGTGTGTGTGTCACACA AATCTGTTAGGTTGG
SP1-utr-mut R	TCGACCAACCTAACAGATTGTGTGTACACACACACA CACACACACACACACACACGAGCT

*F, forward primer; R, reverse primer.

### Cell Lines and Cell Culture

The human RCC cell lines 786-O, ACHN, and Caki-1, as well as one normal kidney cell line HK-2, were purchased from the Shanghai Institute of Cell Biology, Shanghai, China. Cells were cultured in RPMI 1,640 medium with 10% heat-inactivated fetal bovine serum (FBS) under a humidified atmosphere of 5% CO^2^ at 37°C.

### Human Clinical Samples

Twenty-five paired of RCC tissues and adjacent non-tumor tissues were obtained from patients undergoing radical nephrectomy between January 2013 and October 2013 at the First Affiliated Hospital of Medical College, Zhejiang University. The study was approved by the Ethics Committee of Zhejiang University. Informed consent was obtained from each patient in the study. The clinical data of the patients has been listed in our previous studies (Xu et al., 2014). Additionally, a commercial tissue microarray (TMA) bearing 90 pairs of renal cell cancer and corresponding non-tumor tissues was purchased from Shanghai Outdo Biotech to further analyze the expression of miR-362-3p and SP1.

### RNA Isolation and qRT-PCR

RNAiso for Small RNA (TaKaRa, Japan) was used to extract small RNA from clinical samples and cultured cell lines. Then small RNA was reversely transcribed into dsDNA using the One Step PrimeScript miRNA cDNA Synthesis Kit (TaKaRa, Japan). By contrast, total RNA was isolated and transcribed into cDNA using the RNAiso Plus (TaKaRa, Japan) and PrimeScript RT Reagent Kit (TaKaRa, Japan), respectively. ABI 7500 FAST Real-Time PCR System (Applied Biosystems, United States) and SYBR Green PCR Kit (Takara, China) were used for qRT-PCR analysis. The relative expression level was calculated with the 2-ΔΔCt method after normalization with U6 or GAPDH, respectively. The primers are shown in Table 1.

### Dual-Luciferase Reporter Assay

Oligonucleotide pairs that contained the desired miR-362-3p target sequence or mutant sequence were inserted into pmirGLO Dual-Luciferase miRNA Target Expression Vector (Promega, United States), between the *Sac*I and *Sal*I sites. The insertions were confirmed by sequencing. 786-O cells were seeded in 24-well plates and co-transfected with 25 nM miR-362-3p or NC and 100 ng reporter pmirGLO. After 48 h, the relative luciferase activity was measured with Dual-Luciferase Reporter Assay System (Promega, United States).

### Cell Counting Kit-8 (CCK-8) Assay

786-O and ACHN cells were plated in 96-well plates and incubated for overnight. RNA duplex (miR-362-3p, siSP1, or NC) were transfected into the cells with concentration ranging from 10 to 50 nM. After 48 or 72 h, the medium was removed and WST-8 (Dojindo Laboratories, Japan) was added into each well. The absorbance of the solution was measured spectrophotometrically at 450 nM with a MRX II absorbance reader (Dynex Technologies, United States).

### Colony Formation Assay

786-O and ACHN cells were transfected with 2′-O-Methyl modified duplexes (25 nM) for overnight. Then, the cells were trypsinized to a single cell suspension and seeded in 6-well plates (500 cells per well). After culture for 10 days, the colonies were fixed with absolute methanol and stained with 0.1% crystal violet.

### *In vivo* Tumorigenicity Assay

Male BALB/c-nude mice (4 weeks old) were purchased from the Shanghai Experimental Animal Center, Chinese Academy of Sciences, Shanghai, China. 786-O cells (1 × 10^6^ in 100 μl PBS) were injected subcutaneously into the flanks of nude mice. When palpable tumors arose, the mice were injected intratumorally with 30 μg of Lipofectamine 2000-encapsulated miR-362-3p or NC every 3 days. Tumor growth was determined by caliper measurements of two perpendicular diameters, and the volume of the tumor was calculated with the formula V = (width^2^ × length × 0.52). Animal studies were performed according to institutional guidelines.

### Cell Cycle Analysis by Flow Cytometry

Cells were transfected with RNA duplexes (miR-362-3p, siSP1 or NC) for 48 h. Then, the cells were collected and fixed in 75% ethanol overnight at –20°C. After treatment with RNase A and propidium iodide (50 μg/mL) treatment for 30 min, cell cycle analyses were performed with the BD LSRII Flow Cytometer System and FACSDiva Software (BD Bioscience, United States).

### Cell Migration and Invasion Assay

Transwell chambers (Millipore, United States) were used for cell migration and invasion assay. For the invasion assay, the chambers were coated with Matrigel (BD Biosciences, United States) on the upper surface. Cells were transfected with RNA duplexes (miR-362-3p, siSP1 or NC) for 48 h and then 8 × 10^4^ cells suspended in 0.2 mL serum-free medium were added to the chambers. RPMI-1640 with 10% FBS was added to the lower compartment as a chemoattractant. After incubation for 24 h, the cells on the lower surface of the membrane were fixed with absolute methanol and stained with 0.1% crystal violet. Five visual fields of × 200 magnification of each chamber were randomly selected and counted under a light microscope (Olympus, Japan).

### Immunohistochemistry (IHC) Staining

IHC staining was performed as described previously (Chen et al., 2012). Briefly, after the slides were dewaxed, rehydrated, and antigen retrieved, the sections were incubated with anti-SP1 (Proteintech Group Inc., United States) overnight. Then, the slides were incubated with an HRP-conjugated secondary antibody for 1 h. Finally, DAB was used for color development. The strength of positivity was semi-quantified based on the staining intensity and the percentage of positive cells.

### Chromogenic *in situ* Hybridization (CISH)

A 5′-DIG and 3′-DIG labeled, locked nucleic acid-incorporated miRNA probe (miRCURY LNATM Detection probe, Exiqon, Woburn, MA, United States) was used for the detection of miR-362-3p in RCC TMA. The probe sequence was designed as follows: 5′–3′. The specific manipulations were performed as previously described (Liang et al., 2017).

### Western Blot Analysis

Western blot analysis was performed as previously described (Xu et al., 2013) with the following primary immunoblotting antibodies: anti-GAPDH (Sango Biotech, China), anti-p-AKT, anti-p-RB, anti-p-FOXO3 (Epitomics, United States), anti-Snail, anti-CCND1 (Cell Signaling Technology, United States), and anti-SP1 (Proteintech Group Inc., United States).

### Statistical Analysis

The data were expressed as the mean ± SD. All analyses were performed using GraphPad Prism version 5 for Windows and a two-tailed value of *P* < 0.05 was considered statistically significant.

## Results

### miR-362-3p Is Down-Regulated in RCC

**FIGURE 1:**
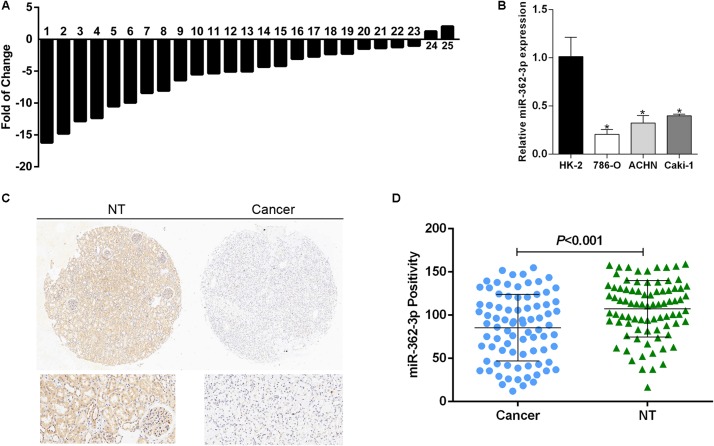
miR-362-3p is frequently downregulated in RCC. **(A)** The relative mRNA expression of miR-362-3p in 25 pairs of RCC tissues were presented as the fold change of miR-362-3p referred to the corresponding adjacent non-tumor tissues. **(B)** Relative expression levels of miR-362-3p in RCC cell lines compared to normal cell line HK-2. **(C)** The representative images of CISH of TMAs. **(D)** The CISH results of TMAs indicated that miR-362-3p was significantly downregulated in RCC tissues than adjacent non-tumor tissues.

To explore the expression pattern of miR-362-3p in RCC, we used quantitative-RT-PCR (qRT-PCR) to measure the expression levels of miR-362-3p in 25 pairs of human RCC tissues and adjacent non-tumor tissues. The relative mRNA expression of miR-362-3p was calculated using U6 RNA as an endogenous control. The results suggested that miR-362-3p was generally expressed at low levels in RCC tumor tissues compared to that in matched non-tumor tissues (Figure 1A, 23 out of 25 exhibited a down-regulated pattern). In addition, three RCC cell lines (786-O, ACHN, and Caki-1) and one normal kidney cell line (HK-2) were also detected by qRT-PCR, and the expression of miR-362-3p in all RCC cell lines was significantly downregulated compared with that in HK-2 cells (Figure 1B). Detection of miR-362-3p expression in RCC TMA with CISH method also demonstrated that miR-362-3p was significantly downregulated in RCC tissues than adjacent non-tumor tissues (Figures 1C,D). Therefore, we speculated that miR-362-3p was a potential tumor suppressor in RCC.

### miR-362-3p Suppresses the Proliferation and Motility of RCC Cells

**FIGURE 2:**
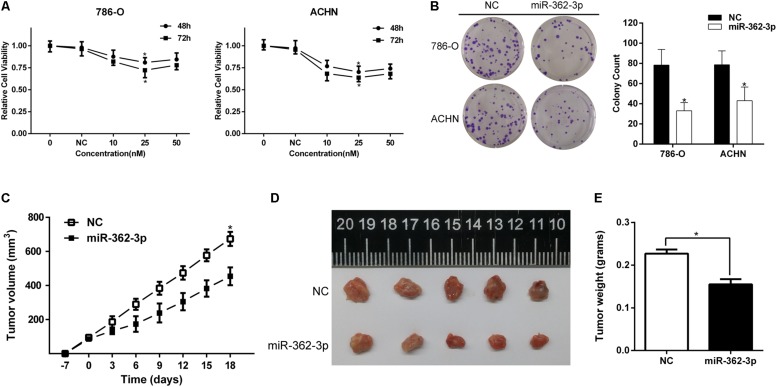
The effect of miR-362-3p on the viability of RCC cells. **(A)** The effect of miR-362-3p on the viability of RCC cells with CCK-8 assay. **(B)** The effect of miR-362-3p on the viability of RCC cells with Colony formation assay (Representative wells were presented). **(C–E)** Xenograft tumor growth assay. The tumor growth curves and tumor weight indicated that xenograft tumor mass in miR-362-3p group was in a significant slower growth pattern, *P < 0.05.

**FIGURE 3:**
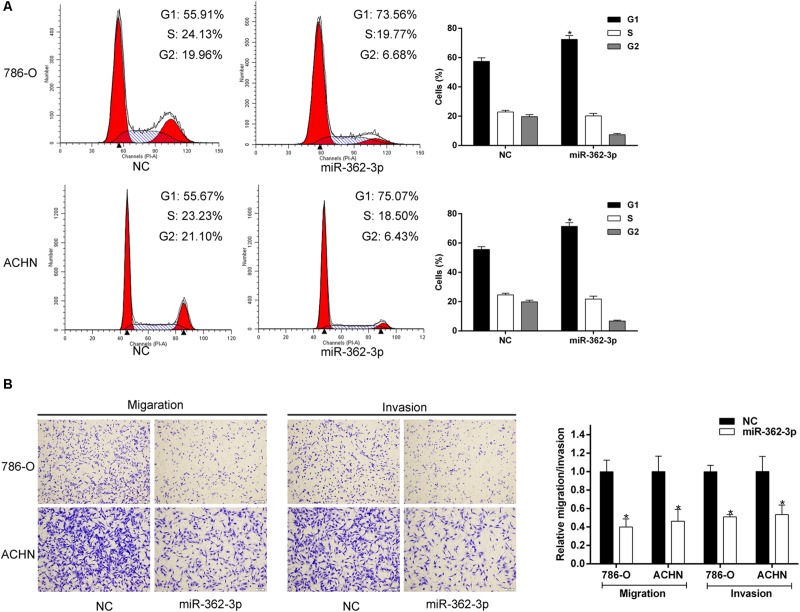
The effect of miR-362-3p on the cell cycle and motility of RCC cells. **(A)** The effect of miR-362-3p on cell cycle progression with Flow cytometric analysis. **(B)** The effect of miR-362-3p on migration and invasion with Transwell assay (representative micrographs were presented), *P < 0.05.

786-O and ACHN cells were transfected with miR-362-3p mimics or NC. Two days after transfection, the fold change of miR-362-3p upregulation was measured with qRT-PCR (Supplementary Figure S1). CCK-8 and colony formation assays suggested that overexpression of miR-362-3p obviously inhibited the proliferation of RCC cells *in vitro*, compared to NC-transfected cells (Figures 2A,B). We then used a xenograft tumor growth assay to explore the effect of miR-362-3p on tumorigenicity *in vivo*. The injection of miR-362-3p mimics into RCC tumor led to a dramatic retardation of tumor growth and weight gain (Figures 2C–E). The impact of miR-362-3p overexpression on cell cycle was explored with flow cytometry. A significant increase in the percentage of cells in the G1/G0 phase, accompanying with a reduction in the percentage of cells in the S phase, was observed in miR-362-3p-overexpressing RCC cells (Figure 3A). We further examined whether miR-362-3p had any effect on RCC migration and invasion capacity using Tanswell chamber assay. As shown in Figure 3B, upregulation of miR-362-3p significantly suppressed the migration and invasion of 786-O and ACHN cells. Overall, the above results indicated that miR-362-3p negatively modulated the proliferation and motility of RCC cells.

### miR-362-3p Regulates the Expression of SP1 by Directly Targeting Its 3′-UTR

**FIGURE 4:**
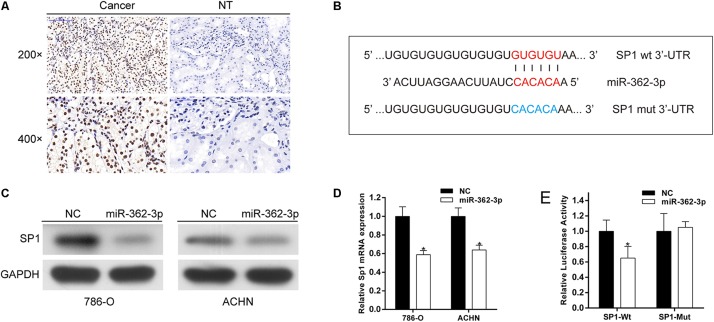
SP1 is a direct target of miR-362-3p. **(A)** IHC analyses of SP1 protein expression. **(B)** The putative seed recognition site in the 3′-UTR of SP1 for miR-362-3p. **(C)** Regulation of SP1 expression by miR-362-3p with Western blot analysis. **(D)** Regulation of SP1 expression by miR-362-3p with qRT-PCR analysis. **(E)** Luciferase reporter assay in HEK-293T cells. miR-362-3p significantly inhibited the luciferase activity of vector that carried 3′-UTR of SP1 but not control vector, *P < 0.05.

A miRNA generally performs its biological function by directly regulate the expression of target genes. SP1, a putative target of miR-362-3p, was identified by bioinformatics prediction. We found that SP1 was commonly overexpressed in RCC tissues compared with that in adjacent non-tumor tissues (Figure 4A). The 3′-UTR of SP1 mRNA has one putative miR-362-3p binding site (Figure 4B). We found that SP1 expression was substantially decreased in both mRNA and protein levels after miR-362-3p overexpression in RCC cells (Figures 4C,D). Finally, we performed luciferase reporter assays to verify a direct interaction between miR-362-3p and the 3′-UTR of SP1. 786-O cells transfected with the 3′-UTR-reporter of SP1 and miR-362-3p showed significantly reduced relative luciferase activity. By contrast, the luciferase activity of the control vector was unchanged by the simultaneous transfection of miR-362-3p (Figure 4E). Taken together, these results suggested that SP1 was a direct target of miR-362-3p.

### Repression of SP1 Phencopied the Effects of miR-362-3p Overexpression in RCC Cells

**FIGURE 5:**
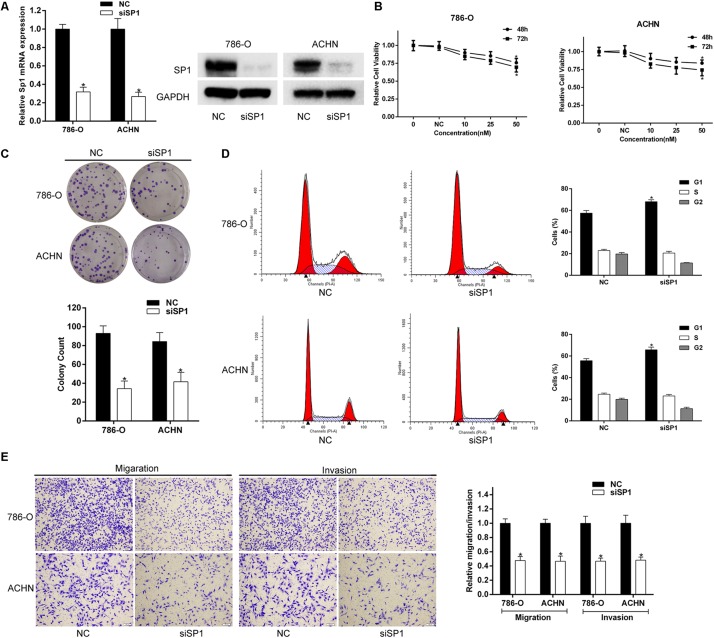
Knockdown of SP1 expression phencopied the effect of miR-362-3p in RCC cells. **(A)** Western blot and qRT-PCR analysis showed reduction of SP1 protein and mRNA expression after siSP1 treatment. **(B)** The effect of siSP1 on the viability of RCC cells with CCK-8 assay. **(C)** The effect of siSP1 on the viability of RCC cells with Colony formation assay (Representative wells were presented). **(D)** The effect of siSP1 on the cell cycle progression with Flow cytometric analysis. **(E)** The effect of siSP1 on migration and invasion with Transwell assay (representative micrographs were presented), *P < 0.05.

The role of SP1 in RCC cells is still not completely clear. We firstly transfected siRNA against SP1 into 786-O and ACHN cells and found a substantially decreased SP1 expression in both protein and mRNA levels (Figure 5A). CCK-8 and colony formation assays suggested that reduction of SP1 expression significantly inhibited the proliferation and tumorigenicity of RCC cells (Figures 5B,C). Co-transfection of a SP1 overexpression vector was applied to abrogate the SP1 expression inhibition by miR-362-3p (Supplementary Figure S2A). Forced SP1 expression partially, but significantly, attenuated the cell viability inhibition induced by miR-362-3p (Supplementary Figure S2B). Flow cytometry showed that repression of SP1 could also significantly induce G1 arrest in RCC cells (Figure 5D). Finally, we explored the role of SP1 in the motility of RCC cells. Transwell chamber assay revealed that the migration and invasion capacity were significantly suppressed in siSP1-transfected cells (Figure 5E). Overall, these data suggested that SP1 was able to regulate proliferation, cell cycle, and motility, which phencopied the effects of miR-362-3p overexpression in RCC cells.

### AKT/FOXO3 Signaling Pathway Is Involved in miR-362-3p-Mediated Inhibition of Cell Proliferation and Motility

**FIGURE 6:**
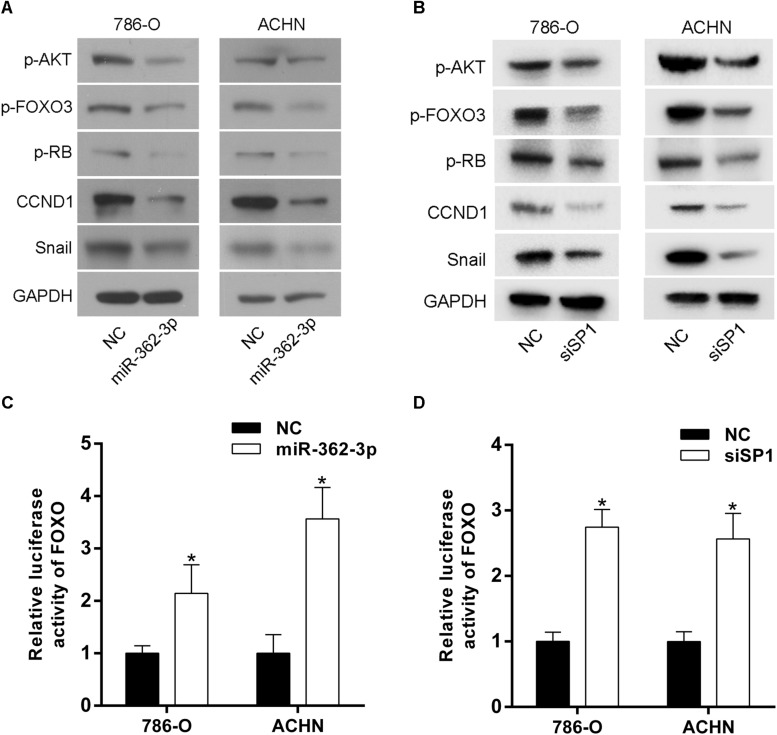
SP1/AKT/FOXO3 signaling pathway mediates the effect of miR-362-3p in RCC cells. **(A,B)** Western blotting analysis demonstrating the effect of miR-362-3p or siSP1 on the protein level of p-AKT, p-FOXO3, p-RB, CCND1 and Snail in RCC cells. **(C,D)** FOXO3 activity was strongly activated by upregulation of miR-362-3p or by knockdown of SP1 expression in RCC cells, *P < 0.05.

AKT/FOXO3 signaling pathway has been reported to play an important role in tumorigenicity and progression of RCC (Ni et al., 2014; Xu et al., 2014). We hypothesized that the upregulation of miR-362-3p might also regulate the AKT/FOXO3 signaling. As shown in Figure 6A, the phosphorylation levels of both AKT and FOXO3 obviously reduced in miR-362-3p-overexpressing RCC cells. The expression of downstream proteins modulated by AKT/FOXO3 signaling, including p-RB, CCND1, and Snail, were obviously decreased. Furthermore, a luciferase reporter vector assay suggested that FOXO3 activity was strongly activated by the overexpression of miR-362-3p (Figure 6C). Considering SP1 was a direct target of miR-362-3p, we then explored whether SP1 mediated the effects of miR-362-3p on AKT/FOXO3 signaling. As shown in Figures 6B,D, the phosphorylation levels of both AKT and FOXO3 were also decreased in SP1-knockdown RCC cells, and FOXO3 activity was strongly induced using luciferase reporter vector assay. Accordingly, the expression levels of p-RB, CCND1, and Snail were substantially decreased. In summary, these results indicated that SP1/AKT/FOXO3 signaling pathway mediated the effect of miR-362-3p on tumorigenicity and progression of RCC.

## Discussion

In recent years, emerging studies have clearly confirmed the important roles of miRNAs in the tumorigenesis of RCC and other malignancies. Although many studies have characterized the miRNA signatures of RCC, the roles of miRNA dysregulation in RCC proliferation and tumorigenicity remain elusive. In the present study, the expression level of miR-362-3p was significantly lower in RCC tissues compared with the corresponding non-tumor tissues. Furthermore, gain-of-function analyses of miR-362-3p *in vitro* and *in vivo* suggest that miR-362-3p was able to suppress the proliferation and metastasis of RCC cells via SP1/AKT/FOXO3 signaling.

miR-362-3p is an intronic miRNA located in intron 3 of the CLCN5 gene on chromosome X. The biological role of miR-362-3p has been investigated in several human cancers. Kang et al. reported that miR-362-3p was downregulated in breast cancer cells because of enhanced DNA methylation. Ectopic expression of miR-362-3p inhibited cell proliferation, migration, and invasion, thereby suppressing tumor growth, by downregulating p130Cas (Kang et al., 2016). Christensen et al. found that miR-362-3p could induce cell cycle arrest and thus reduced cell viability through targeting E2F1, USF2 and PTPN1 and was associated with recurrence of colorectal cancer (Christensen et al., 2013). The study by Assiri et al. suggested that miR-362-3p mediated the transcriptional regulation of hERG and was associated with prognosis in breast cancer (Assiri et al., 2019). By contrast, upregulation of miR-362-3p increased cell proliferation and anchorage-independent soft agar growth by directly targeting Tob2 in hepatocellular carcinoma (Shen et al., 2015). Overexpression of miR-362-3p promoted cell metastasis by suppression of CD82 in gastric cancer (Zhang et al., 2015). MiRNA have been shown to frequently regulate numerous target genes with possible counteracting functions. Thus, the cell context-dependent balance among the network of directly regulated genes of miR-362-3p may determine the biological function in a specific cancer. For RCC, we also cannot rule out the possibility that signaling pathways mediated by other targets, apart from SP1, may play an important role in miR-362-3p-mediated anti-tumor effect.

In this study, SP1 was identified as a novel target of miR-362-3p. SP1 is over-expressed in various cancers and is associated with poor prognosis. Targeting SP1 in cancer treatment has been suggested (Beishline and Azizkhan-Clifford, 2015; Vizcaino et al., 2015). For example, our recent study found that SP1 was overexpressed in bladder cancer and functioned as an oncogene to promote cell migration and invasion (Yan et al., 2018). The role of SP1 in RCC also has been investigated by a few studies. Liu et al. (2017) reported that SP1 could bind to the promoter region of SNHG14 to unregulate its expression in clear cell renal cell carcinoma (ccRCC). Enhanced expression of lncRNA SNHG14 promoted cell migration and invasion by promoting N-WASP protein level. Wu et al. (2016) found that SP1 was a direct target of miR-429, which was able to inhibit cell proliferation, migration, and invasion. All of these findings, as well as our results, supported an oncogenic role for SP1 in RCC.

We also demonstrated that AKT/FOXO3 signaling pathway was regulated by miR-362-3p/SP1 axis. Our previous study (Xu et al., 2014), as well as other researchers’ study (Ni et al., 2014), have found that the transactivation activity of FOXO3 was able to promote cell cycle, cell proliferation, and EMT by reducing the levels of p21Cip1 and p27Kip1, and increases expression of CCND1 and Snail in RCC cells. In the current study, we found that the upregulation of miR-362-3p or downregulation of SP1 decreased AKT phosphorylation and significantly increased the transactivation activity of FOXO3, suggesting that miR-362-3p/SP1/AKT/FOXO3 pathway might represent a new mechanism underlying the development of RCC.

In conclusion, this study suggests that miR-362-3p is an important tumor suppressor in human RCC. miR-362-3p, by regulating SP1/AKT/FOXO3 signaling, is able to inhibit tumorigenesis and progression of RCC cells. The restoration of miR-362-3p could be a potential therapeutic strategy for RCC treatment.

## Data Availability Statement

The datasets generated for this study are available on request to the corresponding author.

## Ethics Statement

The studies involving human participants were reviewed and approved by First Affiliated Hospital, School of Medicine, Zhejiang University. The patients/participants provided their written informed consent to participate in this study. The animal study was reviewed and approved by First Affiliated Hospital, School of Medicine, Zhejiang University.

## Author Contributions

XX, HZ, SW, and HS performed experiments. XX and XZ designed research, analyzed data, and edited the manuscript for intellectual content. All authors read and approved the final manuscript.

## Conflict of Interest

The authors declare that the research was conducted in the absence of any commercial or financial relationships that could be construed as a potential conflict of interest.

## Supplementary Material

FIGURE S1 The ectopic expression of miR-362-3p was confirmed by qRT-PCR, ^∗^*P* < 0.05.

FIGURE S2 Forced expression of SP1 partly rescued miR-362-3p-dependent cell viability inhibition. **(A)** The expression of SP1 or GAPDH was detected by Western blot analysis. **(B)** Forced expression of SP1 partly abrogated anti-tumor effect of miR-362-3p in 786-O cells, ^∗^*P* < 0.05.

## Abbreviations

RCC: renal cell carcinoma
UTR: untranslated region
CCK-8: cell counting kit-8
IHC: immunohistochemistry
miRNA: microRNA
qRT-PCR: quantitative-RT-PCR
siRNA: small interfering RNA
NC: negative control
FACS: fluorescence activated cell sorter.
