# Supplementary figures and images for: SP1/AKT/FOXO3 Signaling Is Involved in miR-362-3p-Mediated Inhibition of Cell-Cycle Pathway and EMT Progression in Renal Cell Carcinoma
Source: Front Cell Dev Biol. 2020 May 5;8:297. doi: 10.3389/fcell.2020.00297 (PMC7214730; doi:10.3389/fcell.2020.00297)

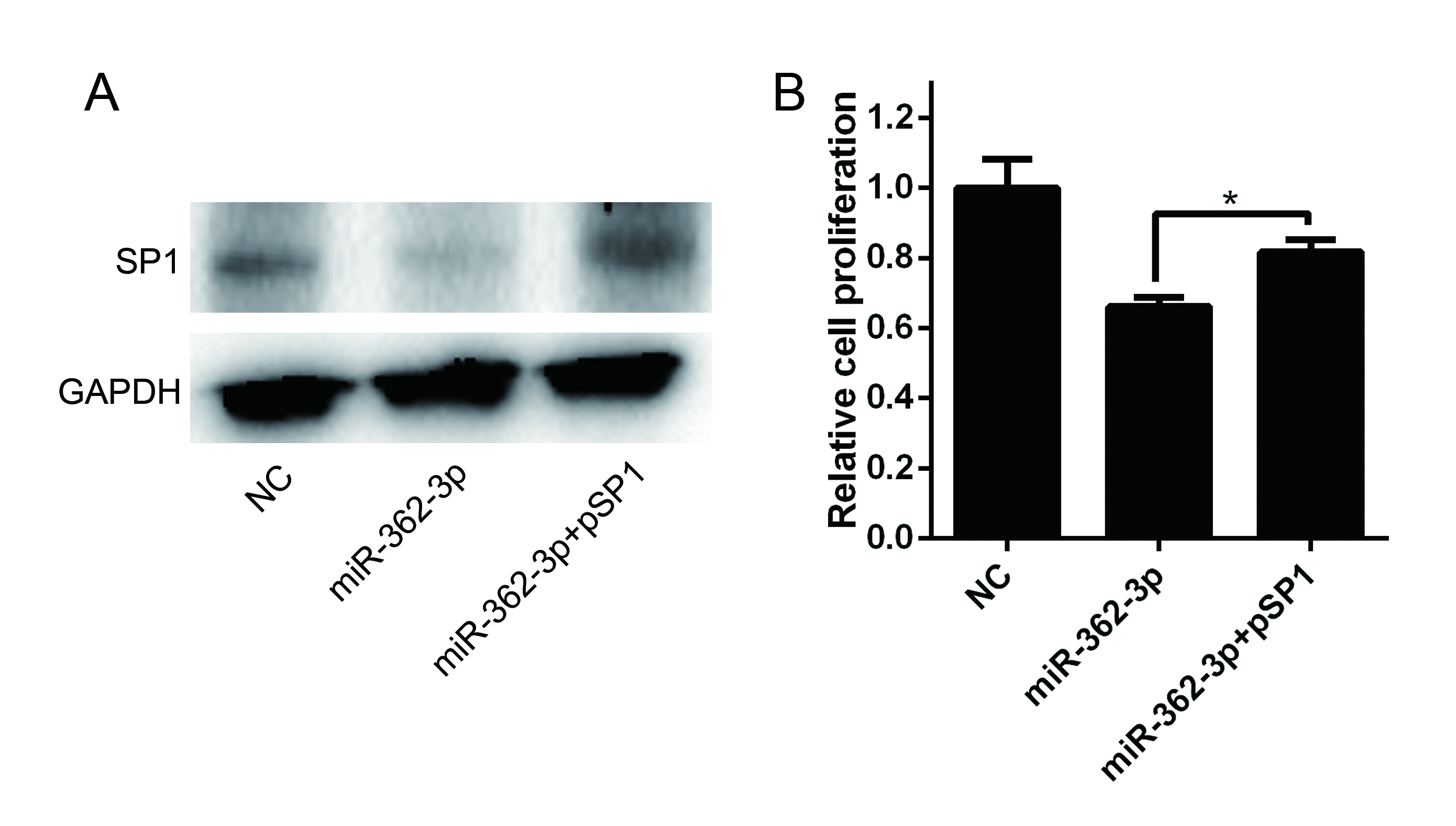

Supplement: FIGURE S2 — Forced expression of SP1 partly rescued miR-362-3p-dependent cell viability inhibition. (A) The expression of SP1 or GAPDH was detected by Western blot analysis. (B) Forced expression of SP1 partly abrogated anti-tumor effect of miR-362-3p in 786-O cells, ∗P < 0.05. [file Image_2.TIF]
